# Enhancement of Heme-Oxygenase 1 in the Injured Peripheral Nerve Following Sulforaphane Administration Fosters Regeneration via Proliferation and Maintenance of Repair Schwann Cells

**DOI:** 10.3390/antiox13091038

**Published:** 2024-08-27

**Authors:** Fabian Szepanowski, Jaqueline Zipfel, Rebecca D. Szepanowski, Bianca Eggert, Nail-Mert Güner, Leon-Phillip Szepanowski, Christoph Kleinschnitz, Anne K. Mausberg, Mark Stettner

**Affiliations:** Department of Neurology and Center for Translational Neuro- and Behavioral Sciences (C-TNBS), University Medicine Essen, University Duisburg-Essen, Hufelandstr. 55, 45147 Essen, Germany

**Keywords:** HO-1, Nrf2, nerve injury, nerve crush, sulforaphane, broccoli extract, neuropathy, Schwann cell, repair Schwann cell, remyelination

## Abstract

Nuclear erythroid 2-related factor 2 (Nrf2) and its downstream effector heme oxygenase 1 (HO-1) are commonly activated in response to cellular stresses. The elevated expression of HO-1 has been associated with markedly accelerated peripheral nerve regeneration. This study aimed to evaluate the impact of a naturally occurring dietary Nrf2/HO-1 activator—sulforaphane (SFN)—on regeneration in a murine sciatic nerve crush model. The beneficial safety profile of SFN has been thoroughly investigated and confirmed several times. Here, SFN was administered daily, starting immediately after C57BL/6 mice were subjected to sciatic nerve crush injury. Injured sciatic nerves were excised at various time points post injury for molecular, immunohistochemical and morphometric analyses. Moreover, functional assessment was performed by grip strength analysis and electrophysiology. Following SFN treatment, the early response to injury includes a modulation of autophagic pathways and marked upregulation of Nrf2/HO-1 expression. This enhancement of HO-1 expression was maintained throughout the regeneration phase and accompanied by a significant increase in repair Schwann cells. In these cells, elevated proliferation rates were observed. Significant improvements in grip strength test performance, nerve conduction velocity and remyelination were also noted following SFN treatment. Collectively, SFN modulates cytoprotective and autophagic pathways in the injured nerve, increasing the number of repair Schwann cells and contributing to effective nerve regeneration. Given the availability of SFN as a nutritional supplement, this compound might constitute a novel regenerative approach with broad patient accessibility and further studies on this topic are warranted.

## 1. Introduction

Peripheral nerve injury causes a partial or total loss of motor, sensory and autonomic functions arising from nerve degeneration distal from the point of injury. The degenerative processes following nerve injury are generally referred to as Wallerian degeneration, characterized by Schwann cell dedifferentiation, inflammation and energy depletion, leading to demyelination and axon disintegration [1,2].

Clinical outcomes following nerve injury or in polyneuropathies exhibit marked heterogeneity, where some patients achieve complete nerve regeneration without significant residual impairments, while others experience only limited recovery [3]. This broad spectrum underscores the potential relevance of substances with pleiotropic effects on nerve regeneration processes.

The initial response to injury occurs within 24 h and is characterized by Schwann cells undergoing dedifferentiation, accompanied by breakdown of the insulating myelin sheaths. Shortly thereafter, axons degenerate from their distal innervating end to the proximal site of injury [2]. Schwann cell dedifferentiation is driven by negative regulators of myelination [4], such as the transcription factors c-Jun and Sox2, which are well characterized [5,6,7]. These negative regulators promote the transition from a myelinating towards a Wallerian phenotype, typically associated with an induction of cytokine and chemokine secretion as well as phagocytic activity. This leads to recruitment and activation of hematogenous and tissue-resident innate immune cells, which—concertedly with dedifferentiated Schwann cells—phagocytize axon and myelin debris [2]. In addition to myelin phagocytosis, emerging lines of evidence suggest that the initial phase of myelin breakdown is characterized by the division of the myelin sheaths into small oval-shaped intracellular fragments, which may be degraded by a selective form of autophagy, termed myelinophagy [8,9]. Thus, phagocytosis and a specific Schwann cell autophagy both contribute to the clearance of myelin debris.

Over the course of nerve regeneration, dedifferentiated Schwann cells adopt a repair phenotype—the so-called “repair Schwann cell”—that provides guidance and trophic support to regenerating axons in bands of Büngner. Negative regulators of myelination such as Sox2 and c-Jun also play important roles in the adoption of this repair phenotype and can, therefore, be considered markers of repair Schwann cells [8,10].

The dynamic transition from a myelinating towards a phagocytic and later repair Schwann cell requires robust molecular defense mechanisms in order to adapt to the rather hostile microenvironment that is present in the injured peripheral nerve. The immense cellular stress exerted by inflammatory cytokines and cytotoxic cues such as reactive oxygen species or necrotic cell debris can only be tolerated through the upregulation of an array of anti-oxidative and anti-inflammatory factors. One central element of this cellular response is the nuclear erythroid 2–related factor 2 (Nrf2) transcription factor, driving the expression of antioxidant response element (ARE) genes [11]. The most versatile downstream effector of Nrf2 signaling may be heme-oxygenase 1 (HO-1) [12]. Besides catalyzing the eponymous enzymatic reaction to remove free heme, HO-1 produces biliverdin, iron ions and carbon monoxide. Biliverdin is subsequently converted to the antioxidant bilirubin. Carbon monoxide is protective through the modulation of metabolic pathways and calcium mobilization. Iron ions are stored in ferritin and can, thus, no longer participate in the Fenton reaction to produce hydroxyl radicals. Therefore, all endproducts of HO-1 can be considered cytoprotective [13,14]. The biological relevance and potency of these enzymatic products is further highlighted by the finding that HO-1 also constitutes a downstream effector of the anti-inflammatory cytokine interleukin-10 [15]. 

In previous work, we have demonstrated by clinical, electrophysiological and morphometric measures that the activation of the Nrf2/HO-1 pathway by the immunomodulatory drug dimethyl fumarate (DMF) effectively promotes nerve regeneration [16]. Nrf2 and HO-1 expression were significantly upregulated after nerve injury, and this cellular response was further enhanced by DMF, resulting in an improvement in nerve regeneration. However, the actual mechanism by which enhanced HO-1 expression resulted in improved nerve regeneration and whether this effect could be achieved by other compounds remained largely elusive. Due to the immunosuppressive effects of DMF and its potential side effects such as gastrointestinal effects and flushes [17], this drug can only be used to a limited extent to promote nerve regeneration. Therefore, we set out to test the therapeutic efficacy of a naturally occurring Nrf2 activator derived from cruciferous plants such as broccoli—sulforaphane (SFN). This investigation aims to ‘mimic’ the regenerative effects of DMF and provide a better understanding of the mechanisms underlying accelerated recovery following enhanced HO-1 induction. Here, we report that sulforaphane modulates cytoprotective and autophagic pathways in the injured nerve, increasing the number of repair Schwann cells and contributing to successful nerve regeneration.

## 2. Materials and Methods

### 2.1. Sciatic Nerve Crush

Male, age-matched (3–4 months) wildtype C57BL/6J mice (Janvier Labs, Le Genest-Saint-Isle, France) were anesthetized for surgery via intraperitoneal injection of a mixture of xylazine (Ceva, Germany) (10 mg/kg) and ketamine (Serumwerk Bernburg, Germany) (100 mg/kg) and placed on a heating plate (37 °C) to maintain constant body temperature. The fur of the lower back was removed with an electric razor, and the skin was disinfected using 70% ethanol. All instruments were sterilized. A small incision (1 cm) was made in the skin above the right hindlimb between the mm. gluteus maximus and biceps femoris. Opening the facial plane between both muscles revealed the sciatic nerve which was carefully lifted using bent forceps and crushed right before its distal branches using a non-serrated clamp at maximum intensity for 30 s. The nerve was replaced under the muscle, and the incision was closed using non-absorbable suture material. The contralateral nerve was not crushed to serve as control. 

### 2.2. Approval for Animal Experimentation

Experimental procedures involving animals were approved by local authorities (LANUV, North Rhine-Westphalia, Germany; approval number 81-02.04.2021.A064) and conducted in adherence to the German Animal Welfare Act and EU guidelines.

### 2.3. Treatment with Sulforaphane (SFN)

Mice received SFN (ApexBio, Houston, TX, USA) at a concentration of 10 mg/kg body weight once daily via intraperitoneal injection, starting immediately after crush injury was introduced. SFN was dissolved in phosphate-buffered saline (Dulbecco’s PBS, sterile, Gibco) containing 10% ethanol (Sigma-Aldrich, Steinheim, Germany). Control animals received an equal volume of vehicle (VEH).

### 2.4. Electrophysiology

Nerve conduction velocities and compound muscle action potentials were determined at 21 days post injury. Mice were anesthetized with a mixture of ketamine (100 mg/kg) and xylazine (10 mg/kg) and immediately placed on a heating plate (37 °C) to maintain constant body temperature. The stimulation of the sciatic nerve was performed by repetitively generated single pulses using monopolar needle electrodes until supramaximal stimulation was achieved. Compound muscle action potential was recorded at the plantar foot muscle with a needle electrode using an electrodiagnostic system (Natus Keypoint). Nerve conduction velocity was calculated from the distance and the motor latency differences between proximal and distal stimulations.

### 2.5. Assessment of Nerve Function by Grip Strength Analysis

Nerve function was evaluated via grip strength analysis of the right (crushed) and left (non-crushed) hindlimbs using a modified force gauge (Erichsen Physimeter 906 MC-B) at 2 days before crush injury and 7, 14 and 21 days post injury. The mouse was handled by the experimenter with a tight grip behind its head, still allowing the hind limbs to move freely. Subsequently, the hind limbs were gently pulled over a metal bar and the maximum force applied before the mouse lost its grip was recorded. Mice were tested three times in succession and data were averaged for each mouse and time point.

### 2.6. Immunohistochemistry

All sections were post-fixed in 4% paraformaldehyde for 20 min. Immunohistochemical procedures were then performed as described previously [18]. Briefly, slides were washed once in PBS and twice in PBT (PBS + 0.1% triton X-100) for 5 min each. Slides were incubated with blocking solution (10% normal goat serum in PBT) for 30 min at room temperature. Primary antibody was applied and slides were incubated at 4 °C overnight. Slides were washed twice for 5 min in PBT, and secondary antibody was applied and incubated at room temperature for 1 h. Slides were washed 5 min in PBT and 5 min in PBS and mounted with Mowiol containing 4,6′diamidino-2-phenylindole (DAPI). Images were acquired using a Leica DMi8 microscope.

### 2.7. Antibodies

The following primary antibodies were used for immunohistochemical applications at the indicated dilutions: rabbit anti-Sox2 (1:250; Abcam #ab97959); rabbit anti-c-Jun (1:100; Cell Signaling #9165); rat anti-Ki-67 (1:100; Invitrogen #14-5698-82); rabbit anti-p-Stat3 (SER727) (1:250; Invitrogen #44-384G); rabbit anti-p75-NTR (1:100; Cell Signaling #8238S). Secondary antibodies donkey anti-rabbit Alexa Fluor 488 (1:250; Invitrogen #A21206) and goat anti-rat Alexa Fluor (1:250, Invitrogen # A A241434) were used. 

For immunoblotting, the following primary antibodies were used: rabbit anti-Heme-oxygenase-1 (1:1000; Abcam; #ab189491); rabbit anti-Beclin-1 (1:1000; Cell Signaling #3495); rabbit anti-LC3 A/B (1:1000; Cell Signaling; #12741); rabbit anti-Glutathione Peroxidase 4 (1:1000; Abcam #ab125066) rabbit anti-ß-Actin (1:10,000; Abcam #ab8227); mouse anti-ß-Actin (1:10,000; Abcam #ab8226). As secondary antibodies peroxidase-conjugated donkey anti-rabbit (1:5000, Jackson ImmunoResearch, #711-035-152) and peroxidase-conjugated donkey anti-mouse 1:5000, Jackson ImmunoResearch, #715-035-150) antibodies were used. 

### 2.8. Sciatic Nerve Histology

Sciatic nerve histology was performed essentially as described previously [18]. Nerves were fixed in 0.1 M cacodylate buffer containing 2.5% glutaraldehyde and kept at 4 °C overnight. The fixative was discarded and replaced by washing buffer (0.1 M cacodylate + 3% sucrose). Nerves were washed for four days at 4 °C. Washing was followed by incubation in an osmium tetroxide reagent for 3 h. Osmium tetroxide reagent was composed of one part 5% potassium dichromate solution (pH 7.4), one part 3.4% NaCl solution and two parts 2% osmium tetroxide solution (Sigma-Aldrich). Afterwards, samples were briefly washed in 0.1 M cacodylate buffer. Samples were dehydrated in an ascending ethanol series (70%; 96%; ≥99.8% undenatured ethanol) for one hour each. Following dehydration, samples were incubated in propylene oxide (Sigma-Aldrich) in tightly closed containers for one hour at room temperature, then one hour in a 1:1 mixture of propylene oxide/epon (epoxy embedding medium kit; Sigma-Aldrich) and finally kept at 4 °C in epon only overnight. Samples were placed in silicone molds and covered with epon embedding mixture. Embedded samples were incubated at 37 °C for 6 h, at 47 °C for 15 h and finally at 60 °C for 28 h until epon was completely hardened. Sectioning was performed approximately 3 mm distal from the crush site. Transverse sections were prepared at a thickness of 1 μm using an ultra-cut microtome and immediately stained with toluidine blue (1% toluidine blue (*w*/*v*) dissolved in a 1% disodium tetraborate (*w*/*v*) solution), washed in distilled H_2_O (approximately 10 mL) containing 1–2 drops of acid ethanol (0.01% HCl in absolute ethanol), placed on a microscope slide, dried on a heating plate and mounted. Sections were photographed on a Leica DMi8 microscope. One representative slide per nerve was evaluated through detailed morphometric assessment.

### 2.9. Assessment of Morphometric Data

Morphometric analysis was performed by a blinded investigator using Fiji ImageJ 1.53c (National Institutes of Health, Bethesda, MA, USA). Axon numbers of whole fascicles were measured manually by marking each individual axon. Axon numbers were normalized to fascicle area. For the evaluation of g-ratios and axonal diameters, the circumference of axons and their respective myelin sheaths was measured within randomly selected fields. A minimum of 200 axons per nerve were evaluated. For the calculation of g-ratios, axonal circumference was divided by the circumference of the respective myelin sheath. Axonal diameters were calculated from the axonal circumference.

### 2.10. Quantification of Malondialdehyde

Sciatic nerve malondialdehyde levels were determined by the thiobarbituric acid method using the commercially available Lipid Peroxidation (MDA) Assay Kit (Sigma-Aldrich). Sciatic nerve samples were homogenized in assay buffer. The detection of MDA was performed as recommended by the manufacturer. Sciatic nerve MDA content was normalized to the respective protein content of the sample.

### 2.11. Immunoblotting

Sciatic nerve tissue was homogenized in radio immunoprecipitation assay (RIPA) buffer (Pierce, Thermo Scientific, Waltham, MA, USA), with protease and phosphatase inhibitor, using ultrasound for 1 min (Thermo Scientific) and stored at −80 °C after centrifugation at 4 °C and 16,000 rpm for 30 min. 

After protein quantification using bicinchoninic acid (BCA) protein assay kit (Pierce, Thermo Scientific), samples were prepared with Laemmli Sample Buffer (BioRad, Feldkirchen, Germany) and boiled at 95 °C for 10 min. Samples with total protein of 15 µg were loaded to 4–15% Mini-PROTEAN^®^ TGX™ Precast Gel and separated in Tris/glycine/SDS running buffer to protein samples by SDS-PAGE (Bio Rad). Samples were transferred to nitrocellulose membranes (BioRad) with Trans-Blot Turbo Transfer System (Bio Rad). Membranes were blocked in EveryBlot Blocking Buffer (Bio Rad). Membranes were incubated with primary antibodies at 4 °C overnight. Further, membranes were washed in tris-buffered saline with Tween. Membranes were incubated with an horse-radish peroxidase coupled antibody and EveryBlocking Buffer for 1 h at room temperature. For chemiluminiscence detection, Clarity Western ECL Substrate was used for 5 min (BioRad). Blots were imaged using the ChemiDoc Imaging System (BioRad). Ponceau S (Sigma-Aldrich) stain was used to visualize total amount of protein. Western blot analysis was performed using ImageJ. Protein signals were normalized to each total protein level and further normalized to actin/total protein value.

### 2.12. RNA Extraction, cDNA Synthesis and Quantitative Polymerase Chain Reaction (qPCR)

Freshly excised sciatic nerves were stored directly in RNAprotect Tissue Reagent (Qiagen, Germany) and stored at −20 °C before further processing. Tissue samples were then transferred to trizol reagent for homogenization using the gentleMACs Dissociator (Miltenyi Biotec, Germany). Following homogenization, RNA was extracted using the phenol–chloroform method. Further, precipitated RNA was analyzed with a NanoDrop One/One spectrophotometer (Thermo Scientific). Samples were adjusted to 50 ng/µL for cDNA synthesis. cDNA was synthesized using the High-Capacity cDNA Reverse Transcription Kit (appliedbiosystems) according to the manufacturer’s protocol. qPCR was subsequently performed in the StepONe Plus Real Time PCR System (Thermo Scientific) using PowerUP SYBR Green Master Mix (Thermo Scientific). Nrf2 primer pairs were ordered from Origene, Germany (Cat. #NW_01902, Origene). Heme Oxygenase-1 (fwd: GTGGAGACGCTTTACATAGTGC; rev: CTTTCAGAAGGGTCAGGTGTCC) [19] and ß-actin (fwd: GAAGTGTGACGTTGACATCCG; rev: TGCTGATCCACATCTGCTGGA) [20] primer pairs were ordered from Eurofins. ß-actin was used as reference and relative expression levels were calculated accordingly.

### 2.13. Image and Data Analysis

An analysis of images was performed using Fiji ImageJ (National Institutes of Health, Bethesda, MA, USA). For the quantification of cell populations, three longitudinal sections per animal were photographed with high magnification (Leica DMi8, Leica, Germany). Cells were counted by a blinded investigator using Fiji ImageJ and either adjusted to the analyzed area or normalized to the total cell count of DAPI-positive cells, respectively. Data analysis and compilation of graphs was carried out using Microsoft (Redmond, WA, USA) Excel 2016 and GraphPad (La Jolla, CA, USA) Prism 7. Statistical analysis was conducted using Student’s *t*-test; non-parametric data were analyzed by Mann–Whitney test. Statistical significance is indicated by asterisks with * *p* ≤ 0.05, ** *p* ≤ 0.01, and *** *p* ≤ 0.001.

## 3. Results

### 3.1. Sulforaphane Promotes Nerve Regeneration through an Improvement in Remyelination

**Figure 1:**
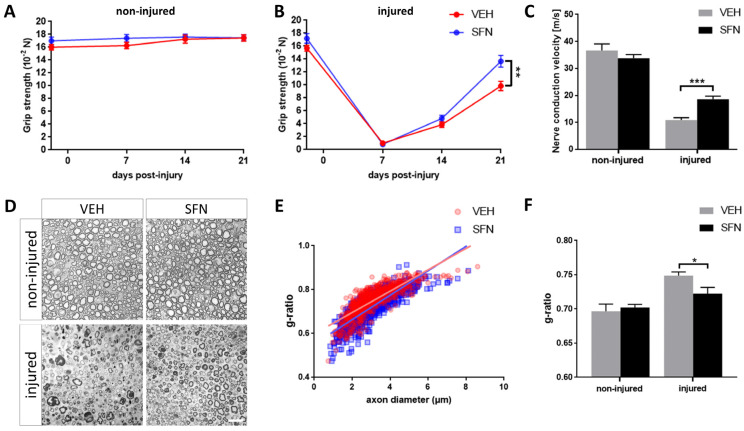
Sulforaphane promotes nerve regeneration by clinical, electrophysiological and histomorphometric measures. Grip strength test was performed in the uninjured (**A**) and injured (**B**) hindlimbs at 2 days before and 7, 14 and 21 days post injury (*N* = 18/18 for both groups). Nerve conduction velocity (**C**) was assessed as final endpoint at 21 days post injury (N = 18/17/18/17 from left to right). Representative images of semi-thin sections obtained at day 21 post injury (**D**) were analyzed to assess morphometric parameters. Scale bar indicates 20 µm. G-ratios were plotted against axon diameters (**E**), suggesting a shift towards increased myelin thickness without affecting axonal diameter distribution. Equations for linear regression lines (VEH: Y = 0.04872X + 0.5984; SFN = Y = 0.05569X + 0.552). G-ratio measurements (**F**) indicated a significant improvement in myelin thickness at 21 days post injury with SFN versus VEH (N = 4/4 for both groups). Statistical significance is indicated by asterisks with * *p* ≤ 0.05, ** *p* ≤ 0.01 and *** *p* ≤ 0.001. Data represent mean ± s.e.m.

We performed unilateral sciatic nerve crush injury in C57BL/6 mice subjected to daily treatment with the plant-derived isothiocyanate sulforaphane (SFN). Clinical assessments were conducted through grip strength analysis of both the injured and uninjured hindlimbs at various intervals: 2 days before and 7, 14 and 21 days post injury (Figure 1A,B). While grip strength in the injured hindlimb was nearly lost at day 7, mice gradually recovered over the subsequent two weeks. By day 21, the SFN-treated group exhibited significantly enhanced grip strength (Figure 1B). This clinical finding was further corroborated by electroneurographic recordings, which revealed notably increased nerve conduction velocity in the crushed nerves after 21 days of SFN treatment (Figure 1C). To investigate histomorphometric parameters, semi-thin nerve sections were analyzed on day 21 to evaluate axon diameters and g-ratios, the ratio between axonal and whole myelinated fiber diameter (Figure 1D). Reflecting the clinical and electrodiagnostic improvements, SFN-treated mice showed significantly enhanced myelin thickness, while the overall distribution of myelinated axons remained unchanged (Figure 1D–F; Supplementary Figure S1).

### 3.2. Sulforaphane Amplifies Nrf2/HO-1 Expression and May Modulate Beclin-Independent Autophagy in the Early Response to Injury

**Figure 2:**
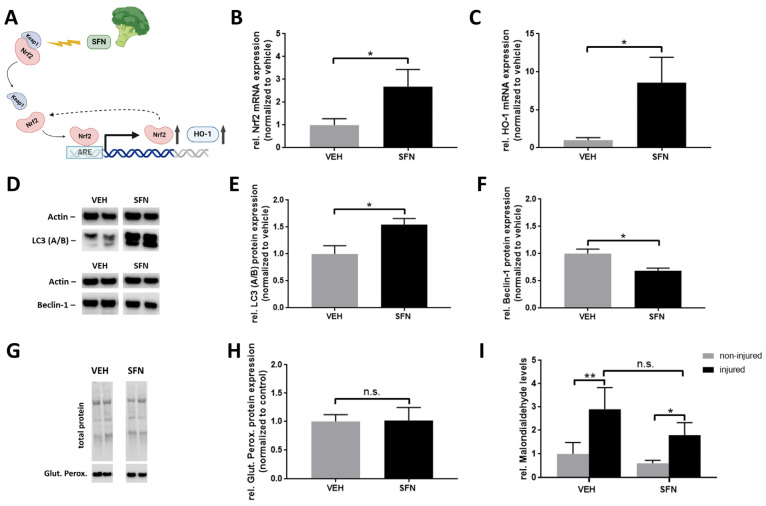
Sulforaphane amplifies Nrf2/HO-1 and may increase Beclin-independent autophagy in the early response to injury. (**A**) Schematic of SFN-mediated Nrf2 induction. SFN interferes with the Nrf2 repressor Keap1, enabling translocation of Nrf2 into the nucleus to drive antioxidant response element (ARE) gene expression. This includes the downstream effector HO-1 while simultaneously promoting Nrf2 expression in a positive feedback loop. Quantification of (**B**) Nrf2 and (**C**) HO-1 expression in the injured nerve 7 days post injury via qPCR (*N* = 4/4 per group). Representative immunoblots (**D**) and quantification of autophagy-related proteins (**E**) LC3 (A/B) (14 kDa (cytosolic form)/16 kDa (lipidated form)) (*N* = 7/7) and (**F**) Beclin-1 (52 kDa) (*N* = 7/5). Representative immunoblot (**G**) and quantification (**H**) of glutathione peroxidase 4 (20 kDa) (*N* = 7/7) in the injured nerve at 7 days post injury. Quantification of the lipid peroxidation marker malondialdehyde in the uninjured and injured nerve (N = 13/13/14/14 from left to right) (**I**). Statistical significance is indicated by asterisks with * *p* ≤ 0.05, ** *p* ≤ 0.01. Data represent mean ± s.e.m.

In order to better understand the molecular mechanisms underlying enhanced nerve regeneration, we conducted an analysis of key proteins and pathways potentially influenced by SFN in the early response to injury. At day 7 post injury, we began by examining the expression of Nrf2 and its downstream target HO-1 using quantitative PCR in the injured nerve. Both Nrf2 and HO-1 mRNA expression showed a substantial increase following SFN treatment (Figure 2A–C). Recognizing the vital role of Schwann cells in early myelin debris clearance, we evaluated markers of autophagic pathways. We observed a marked elevation in both cytosolic and lipidated forms of microtubule-associated protein 1A/1B-light chain 3 (LC-3 (A/B)) (Figure 2D,E) expression, coinciding with a significant reduction in Beclin-1 protein levels (Figure 2D,F), suggesting a possible increase in Beclin-independent autophagy. Parameters related to oxidative defense mechanisms remained largely unaffected, with no notable changes in the protein expression of glutathione peroxidase (Figure 2G,H). Similarly, malondialdehyde, a marker of lipid peroxidation, exhibited a discrete but statistically insignificant reduction under SFN treatment (Figure 2I).

### 3.3. Sustained Engagement of HO-1 Expression Is Associated with Maintenance and Proliferation of Repair Schwann Cells 

**Figure 3:**
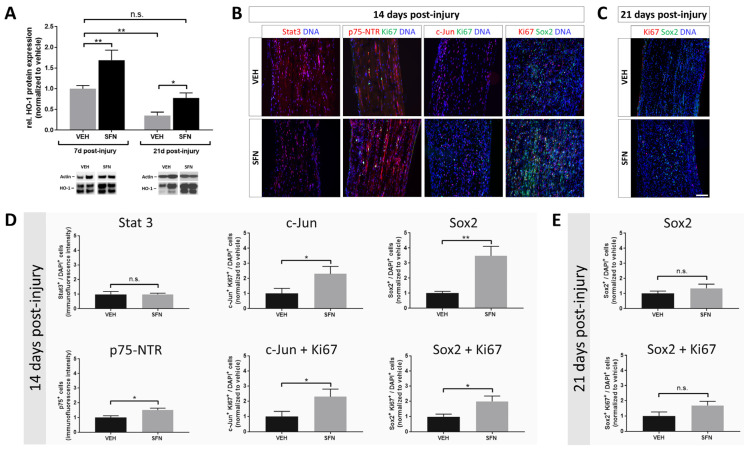
Sustained expression of HO-1 following sulforaphane is associated with the maintenance and proliferation of repair Schwann cells. (**A**) Representative immunoblots and quantification of HO-1 (28–33 kDa) at 7 and 21 days post injury (N = 6/7/7/8 from left to right). (**B**) Representative images of immunofluorescence staining of repair Schwann cell markers Stat3, p75-NTR, c-Jun and Sox2 and the proliferation marker Ki67 at 14 post injury. (**C**) Representative images of Sox2 and Ki67 at 21 days post injury. (**D**) Quantification of the respective markers at 14 days (Stat3—*N* = 6/6; p75-NTR—*N* = 5/5; c-Jun—*N* = 8/8; c-Jun+Ki67—*N* = 6/6; Sox2—*N* = 6/7; Sox2+Ki67—*N* = 8/8) and (**E**) 21 days (Sox2—*N* = 9/8; Sox2+Ki67—*N* = 9/8) post injury. Statistical significance is indicated by asterisks with * *p* ≤ 0.05, ** *p* ≤ 0.01. n.s. no significant difference. Data represent mean ± s.e.m.

To gain a deeper understanding of the factors contributing to the observed improvement beyond the early response to injury, we evaluated the expression of the HO-1 protein in the injured nerve at 7 and 21 days post injury (Figure 3A). We found that SFN-treated animals showed a persistent expression of HO-1 throughout the course of nerve regeneration. Notably, while HO-1 levels declined significantly in both groups by day 21 post injury, the SFN-treated mice exhibited a slower decrease in HO-1 expression compared to the vehicle-treated group, indicating that SFN treatment helps maintain higher HO-1 levels over time (Figure 3A). 

At 14 days post injury, marking an intermediate phase in the ongoing process of nerve regeneration, we assessed the expression of potential markers for repair Schwann cells—specifically Stat3, p75-NTR, c-Jun, and Sox2 (Figure 3B,D). With the exception of Stat3, all markers demonstrated a substantial rise in the population of repair Schwann cells, with the greatest increase observed for Sox2-positive cells (Figure 3D). Notably, c-Jun and Sox2-positive cells exhibited considerable co-staining with Ki67 (Figure 3D), implying that HO-1 supports both the survival and expansion of repair Schwann cells. By day 21, Sox2-positive cells, serving as representative repair Schwann cell marker, had largely returned to levels consistent with those observed in the vehicle-treated group (Figure 3C,E). Nevertheless, they continued to exhibit a modest, albeit non-significant, increase in proliferation (Figure 3C,E).

## 4. Discussion

In this work, we provide in vivo evidence for the regenerative potency of sulforaphane, an isothiocyanate from cruciferous plants such as broccoli, for peripheral nerve injury. This effect appears to be predominantly based on the ability of SFN to activate the Nrf2 transcription factor and its versatile downstream effector, HO-1, in cells of the peripheral nerve, in particular Schwann cells. With regard to translational implications, we chose a dosage of SFN in our mouse model that corresponds to a human equivalent dose of approximately 50–100 mg per day. This dosage of SFN is well achievable with commercially available dietary supplements containing ingredients such as SFN-enriched broccoli extract [21]. As an electrophilic compound, the efficacy and safety of SFN are rooted in the concept of hormesis [22], which emphasizes the importance of low dosing. At low to moderate doses, SFN activates protective pathways, such as the Nrf2 signaling pathway, enhancing cellular resilience and function. However, high doses can cause oxidative stress and toxicity [22]. Thus, optimizing the dosing of SFN is crucial to harness its health benefits and avoid adverse effects. Sulforaphane has demonstrated a favorable safety profile in various studies, showing low toxicity and minimal side effects at therapeutic doses. It is generally considered safe for human consumption, with adverse effects being rare and usually mild, such as gastrointestinal discomfort. Clinical trials have further supported its safety, making it a promising candidate for long-term use as a potential regenerative therapy [23,24].

The regenerative benefits of Nrf2/HO-1 activation in the peripheral nerve were previously established in a study using dimethyl fumarate (DMF) [16]. In this study, we identified an induction of Nrf2 and HO-1 in response to injury, which was further amplified by DMF treatment, resulting in accelerated nerve regeneration. Comparable to DMF treatment, SFN also led to a marked elevation of Nrf2 and HO-1 gene expression, which was evidently translated into increased HO-1 protein expression. Of note, we detected two bands of HO-1, indicating a 33 kDa and a 28 kDa form. While the 33 kDa form is considered the enzymatically active form, the truncated 28 kDa form may be translocated into the nucleus and even further propagate Nrf2/HO-1 expression [25,26]. In contrast to our previous study [16] that only investigated HO-1 expression in the early response to injury, we now report that the stimulation of HO-1 expression is largely maintained throughout the entire regeneration phase up to 21 days post injury. This appears of particular interest, as Nrf2 knockout mice display reduced myelin clearance in line with impaired remyelination and functional recovery [27], suggesting that Nrf2/HO-1 signaling significantly contributes to the adaptation and resistance of Schwann cells to degenerative conditions. However, similar to the effects of DMF [16], SFN did only discretely affect measures of oxidative damage, as shown by the assessment of the lipid peroxidation marker malondialdehyde. These findings suggest that the regenerative effects of Nrf2/HO-1 signaling may involve mechanistically intricate processes beyond merely facilitating oxidative defense mechanisms.

While HO-1 is generally regarded as cytoprotective and anti-inflammatory factor, it has also been linked to heightened autophagic activity in both cell culture and animal models [28,29,30]. Schwann cells are known to play a crucial role in the phagocytosis of myelin debris [31], clearly suggesting an important contribution of autophagic pathways to this function. Interestingly, Schwann cells have been found to induce HO-1 expression as early as 2–3 days after nerve injury, in parallel to the onset of their phagocytic activity [32]. However, evidence regarding specific details on autophagy in Schwann cells is scarce. A study by Gomez-Sanchez et al. [9] provided a more comprehensive insight into the role of Schwann cell-specific autophagic pathways in the context of nerve injury and coined the term ‘myelinophagy’, as autophagic processes in these cells show distinctive features in relation to the removal of myelin debris. Most interestingly, however, this study also provided evidence that the generation of repair Schwann cells is impaired by the use of pharmacological autophagy blockade [9]. These findings align with our observation of increased repair Schwann cell proliferation and maintenance in relation to an elevated expression of the autophagic marker LC3. Both the cytosolic (LC3-I) and lipidated (LC3-II) forms of LC3 were found to be increased, suggesting that not only expression of LC3 may be increased, but also autophagic flux [33]. Interestingly, Gomez-Sanchez et al. [9] reported an increase in Beclin-dependent autophagy, whereas we find Beclin protein expression to be decreased under SFN treatment. In combination with increased LC3 expression, our findings also hint at an important role of Beclin-independent autophagy in the generation or function of repair Schwann cells. The actual meaning of this differential expression pattern of autophagic proteins remains unclear and warrants further investigation.

Nevertheless, the role of Schwann cells in the initial response to injury is becoming progressively clearer, whereas the function of repair Schwann cells during the late phase of nerve regeneration remains less well elucidated. An elegant study, however, demonstrated that the highly elongated repair Schwann cells can undergo significant shortening rather than being removed by the induction of cell death mechanisms [10]. This indicates that repair Schwann cells are not lost but may rather transdifferentiate into myelin-forming Schwann cells. Therefore, an initially larger pool of functionally intact repair Schwann cells could ultimately contribute to a substantially increased number of remyelinating Schwann cells, which may partially explain the impact of elevated HO-1 expression on remyelination.

A limitation of our study is the rather correlative nature of our findings. For instance, HO-1 or autophagy inhibitors could provide more definitive evidence regarding the necessity for a direct requirement of these pathways in enhancing the generation of repair Schwann cells and subsequently improved nerve regeneration. However, the value of employing gene-targeted animals as conceptual validation may be rather questionable. To exemplify, Nrf2-deficient mice display significantly reduced resistance to stressors commonly associated with nerve injury. Consequently, the regenerative processes in these mice are unlikely to be comparable to those observed in wildtype mice treated with SFN [27]. Nevertheless, further mechanistic investigations are crucial to elucidate the precise relationship between HO-1 induction, autophagy, and the formation of repair Schwann cells.

Given the ubiquitous expression and versatile actions of HO-1, our findings suggest that SFN may also be beneficial for neuropathies in general. As a downstream effector of IL-10 [15], the protective and regenerative potency of HO-1 may also apply to inflammatory neuropathies in particular. The modulation of HO-1 expression may shift the polarization of T-lymphocytes (TH1/TH2) and macrophages (M1/M2) towards the protective TH2 and M2 phenotypes, respectively, limiting pro-inflammatory immune responses. In this regard, DMF has been reported to ameliorate the clinical course of experimental autoimmune neuritis (EAN) by causing M2 macrophage polarization via a Nrf2/HO-1 dependent mechanism [34]. In addition, Schwann cells expressing markers such as Sox2 are abundantly present in EAN [35]. While these Sox2-positive Schwann cells are considered dedifferentiating rather than repair Schwann cells [36], the actual nature and function of these cells remains to be elucidated. Thus, it is conceivable that a rectification of the endoneurial milieu by pleiotropic protective factors such as HO-1 may both ameliorate neuroinflammation by addressing immune cell polarization while simultaneously supporting the generation of repair Schwann cells.

In conclusion, our study highlights the regenerative potential of SFN in peripheral nerve injury, primarily via the activation of Nrf2 and HO-1. SFN sustains the Nrf2/HO-1 pathway, promoting nerve regeneration and facilitating Schwann cell functions, which may include survival, proliferation and autophagy for myelin debris clearance. These findings suggest that SFN could serve as a valuable therapeutic approach for addressing peripheral nerve injuries, neuropathies and inflammatory neuropathies, potentially offering renewed prospects for patients contending with these debilitating conditions. These data may pave the way for clinical trials targeting these disorders.

## Data Availability

The original contributions presented in the study are included in the article/supplementary material, and further inquiries can be directed to the corresponding authors.

## Supplementary Materials

The following supporting information can be downloaded at: https://www.mdpi.com/article/10.3390/antiox13091038/s1, Figure S1: SFN does not affect total number of axons or the distribution of myelinated axons at 21 days post injury. (A) The number of axons was assessed in semi-thin sections. All axons of one fascicle were counted and normalized to fascicle area. Axonal density was further normalized to VEH. (B) The distribution of myelinated axons in the regenerating nerve sorted by axon caliber was not affected by SFN. *N* = 4/4 per group. Data represent mean ± s.e.m.

## References

[1] Coleman M.P., Freeman M.R. (2010). Wallerian degeneration, wld^S^, and nmnat. Annu. Rev. Neurosci..

[2] Gaudet A.D., Popovich P.G., Ramer M.S. (2011). Wallerian degeneration: Gaining perspective on inflammatory events after peripheral nerve injury. J. Neuroinflamm..

[3] Faroni A., Mobasseri S.A., Kingham P.J., Reid A.J. (2015). Peripheral nerve regeneration: Experimental strategies and future perspectives. Adv. Drug Deliv. Rev..

[4] Jessen K.R., Mirsky R. (2008). Negative regulation of myelination: Relevance for development, injury, and demyelinating disease. Glia.

[5] Parkinson D.B., Bhaskaran A., Arthur-Farraj P., Noon L.A., Woodhoo A., Lloyd A.C., Feltri M.L., Wrabetz L., Behrens A., Mirsky R. (2008). c-Jun is a negative regulator of myelination. J. Cell Biol..

[6] Roberts S.L., Dun X.P., Doddrell R.D.S., Mindos T., Drake L.K., Onaitis M.W., Florio F., Quattrini A., Lloyd A.C., D’Antonio M. (2017). Sox2 expression in Schwann cells inhibits myelination in vivo and induces influx of macrophages to the nerve. Development.

[7] Le N., Nagarajan R., Wang J.Y., Araki T., Schmidt R.E., Milbrandt J. (2005). Analysis of congenital hypomyelinating Egr2^Lo/Lo^ nerves identifies Sox2 as an inhibitor of Schwann cell differentiation and myelination. Proc. Natl. Acad. Sci. USA.

[8] Jessen K.R., Mirsky R. (2016). The repair Schwann cell and its function in regenerating nerves. J. Physiol..

[9] Gomez-Sanchez J.A., Carty L., Iruarrizaga-Lejarreta M., Palomo-Irigoyen M., Varela-Rey M., Griffith M., Hantke J., Macias-Camara N., Azkargorta M., Aurrekoetxea I. (2015). Schwann cell autophagy, myelinophagy, initiates myelin clearance from injured nerves. J. Cell Biol..

[10] Gomez-Sanchez J.A., Pilch K.S., van der Lans M., Fazal S.V., Benito C., Wagstaff L.J., Mirsky R., Jessen K.R. (2017). After Nerve Injury, Lineage Tracing Shows That Myelin and Remak Schwann Cells Elongate Extensively and Branch to Form Repair Schwann Cells, Which Shorten Radically on Remyelination. J. Neurosci..

[11] Ngo V., Duennwald M.L. (2022). Nrf2 and Oxidative Stress: A General Overview of Mechanisms and Implications in Human Disease. Antioxidants.

[12] Alam J., Stewart D., Touchard C., Boinapally S., Choi A.M., Cook J.L. (1999). Nrf2, a Cap‘n’Collar transcription factor, regulates induction of the heme oxygenase-1 gene. J. Biol. Chem..

[13] Otterbein L.E., Soares M.P., Yamashita K., Bach F.H. (2003). Heme oxygenase-1: Unleashing the protective properties of heme. Trends Immunol..

[14] Soares M.P., Bach F.H. (2009). Heme oxygenase-1: From biology to therapeutic potential. Trends Mol. Med..

[15] Lee T.S., Chau L.Y. (2002). Heme oxygenase-1 mediates the anti-inflammatory effect of interleukin-10 in mice. Nat. Med..

[16] Szepanowski F., Donaldson D.M., Hartung H.P., Mausberg A.K., Kleinschnitz C., Kieseier B.C., Stettner M. (2017). Dimethyl fumarate accelerates peripheral nerve regeneration via activation of the anti-inflammatory and cytoprotective Nrf2/HO-1 signaling pathway. Acta Neuropathol..

[17] Liang G., Chai J., Ng H.S., Tremlett H. (2020). Safety of dimethyl fumarate for multiple sclerosis: A systematic review and meta-analysis. Mult. Scler. Relat. Disord..

[18] Szepanowski F., Derksen A., Steiner I., Meyer Zu Horste G., Daldrup T., Hartung H.P., Kieseier B.C. (2016). Fingolimod promotes peripheral nerve regeneration via modulation of lysophospholipid signaling. J. Neuroinflamm..

[19] Krause D., Suh H.S., Tarassishin L., Cui Q.L., Durafourt B.A., Choi N., Bauman A., Cosenza-Nashat M., Antel J.P., Zhao M.L. (2011). The tryptophan metabolite 3-hydroxyanthranilic acid plays anti-inflammatory and neuroprotective roles during inflammation: Role of hemeoxygenase-1. Am. J. Pathol..

[20] Wang A.L., Niu Q., Shi N., Wang J., Jia X.F., Lian H.F., Liu Z., Liu C.X. (2015). Glutamine ameliorates intestinal ischemia-reperfusion Injury in rats by activating the Nrf2/Are signaling pathway. Int. J. Clin. Exp. Pathol..

[21] Houghton C.A. (2019). Sulforaphane: Its “Coming of Age” as a Clinically Relevant Nutraceutical in the Prevention and Treatment of Chronic Disease. Oxid. Med. Cell. Longev..

[22] Scuto M., Rampulla F., Reali G.M., Spano S.M., Trovato Salinaro A., Calabrese V. (2024). Hormetic Nutrition and Redox Regulation in Gut-Brain Axis Disorders. Antioxidants.

[23] Kensler T.W., Egner P.A., Agyeman A.S., Visvanathan K., Groopman J.D., Chen J.G., Chen T.Y., Fahey J.W., Talalay P. (2013). Keap1-nrf2 signaling: A target for cancer prevention by sulforaphane. Top. Curr. Chem..

[24] Mangla B., Javed S., Sultan M.H., Kumar P., Kohli K., Najmi A., Alhazmi H.A., Al Bratty M., Ahsan W. (2021). Sulforaphane: A review of its therapeutic potentials, advances in its nanodelivery, recent patents, and clinical trials. Phytother. Res..

[25] Biswas C., Shah N., Muthu M., La P., Fernando A.P., Sengupta S., Yang G., Dennery P.A. (2014). Nuclear heme oxygenase-1 (HO-1) modulates subcellular distribution and activation of Nrf2, impacting metabolic and anti-oxidant defenses. J. Biol. Chem..

[26] Yang Q., Wang W. (2022). The Nuclear Translocation of Heme Oxygenase-1 in Human Diseases. Front. Cell Dev. Biol..

[27] Zhang L., Johnson D., Johnson J.A. (2013). Deletion of Nrf2 impairs functional recovery, reduces clearance of myelin debris and decreases axonal remyelination after peripheral nerve injury. Neurobiol. Dis..

[28] Tracey N., Creedon H., Kemp A.J., Culley J., Muir M., Klinowska T., Brunton V.G. (2020). HO-1 drives autophagy as a mechanism of resistance against HER2-targeted therapies. Breast Cancer Res. Treat..

[29] Zou L., Lei H., Shen J., Liu X., Zhang X., Wu L., Hao J., Jiang W., Hu Z. (2020). HO-1 induced autophagy protects against IL-1 beta-mediated apoptosis in human nucleus pulposus cells by inhibiting NF-kappaB. Aging.

[30] Di Tu Q., Jin J., Hu X., Ren Y., Zhao L., He Q. (2020). Curcumin Improves the Renal Autophagy in Rat Experimental Membranous Nephropathy via Regulating the PI3K/AKT/mTOR and Nrf2/HO-1 Signaling Pathways. Biomed. Res. Int..

[31] Nocera G., Jacob C. (2020). Mechanisms of Schwann cell plasticity involved in peripheral nerve repair after injury. Cell. Mol. Life Sci..

[32] Hirata K., He J.W., Kuraoka A., Omata Y., Hirata M., Islam A.T., Noguchi M., Kawabuchi M. (2000). Heme oxygenase1 (HSP-32) is induced in myelin-phagocytosing Schwann cells of injured sciatic nerves in the rat. Eur. J. Neurosci..

[33] Ueno T., Komatsu M. (2020). Monitoring Autophagy Flux and Activity: Principles and Applications. Bioessays.

[34] Han R., Xiao J., Zhai H., Hao J. (2016). Dimethyl fumarate attenuates experimental autoimmune neuritis through the nuclear factor erythroid-derived 2-related factor 2/hemoxygenase-1 pathway by altering the balance of M1/M2 macrophages. J. Neuroinflamm..

[35] Szepanowski F., Winkelhausen M., Steubing R.D., Mausberg A.K., Kleinschnitz C., Stettner M. (2021). LPA(1) signaling drives Schwann cell dedifferentiation in experimental autoimmune neuritis. J. Neuroinflamm..

[36] Szepanowski F., Stettner M. (2023). Focus on LPA signaling: A promising therapeutic target to foster regeneration in immune-mediated neuropathies. Neural Regen. Res..

