# Supplementary material for: Enhancement of Heme-Oxygenase 1 in the Injured Peripheral Nerve Following Sulforaphane Administration Fosters Regeneration via Proliferation and Maintenance of Repair Schwann Cells
Source: Antioxidants (Basel). 2024 Aug 27;13(9):1038. doi: 10.3390/antiox13091038 (PMC11428888; doi:10.3390/antiox13091038)
Supplement: Supplementary file 1 [file antioxidants-13-01038-s001.zip › antioxidants-3136087-supplementary.pdf]

Szepanowski et al.

Enhancement of Heme-Oxygenase 1 in the injured peripheral nerve following sulforaphane administration fosters regeneration via proliferation and maintenance of repair Schwann cells

Supplementary Information

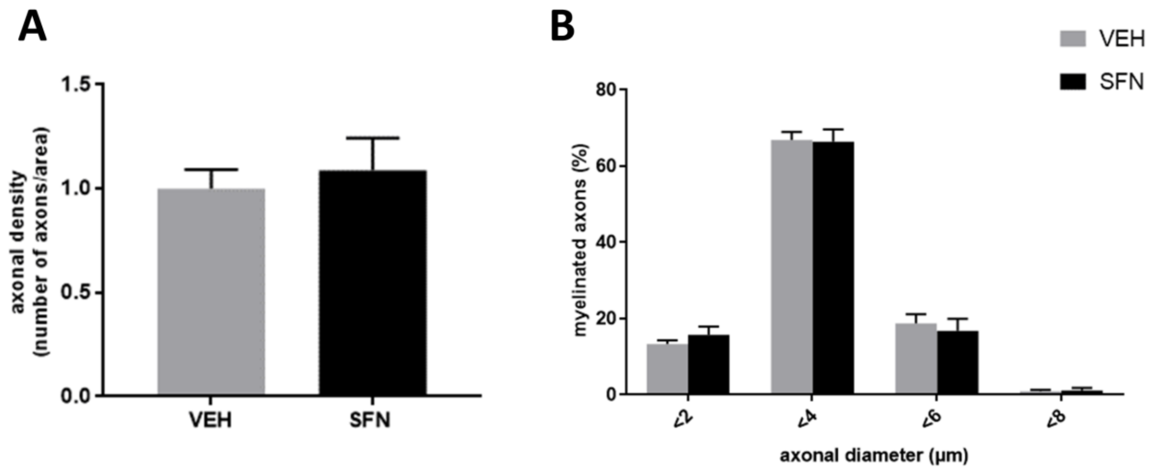

Supplementary Figure S1: SFN does not affect total number of axons or the distribution of myelinated axons at 21 days post-injury. (A) The number of axons was assessed in semi-thin sections. All axons of one fascicle were counted and normalized to fascicle area. Axonal density was further normalized to VEH (B) The distribution of myelinated axons in the regenerating nerve sorted by axon caliber was not affected by SFN. *N = 4/4* per group. Data represent mean ± s.e.m.
